# PK-PD correlation of anti-infective agents for dose adjustment in one severe burn child with sepsis

**DOI:** 10.1186/cc10174

**Published:** 2011-06-22

**Authors:** EV Campos, DS Gomez, RP Azevedo, A Despinoy, MC Ferreira, FF Souza, C Vieira, CS Giraud, SRCJ Santos

**Affiliations:** 1 Hospital das Clinicas da Faculdade de Medicina da Universidade de São Paulo, São Paulo - SP, Brazil; 2 School of Pharmaceutical Sciences, University of São Paulo, São Paulo - SP, Brazil

## Introduction

Altered pharmacokinetics in patients with major burns may result in anti-infective plasma concentrations below those required to be effective against the common pathogens encountered in burn patients. Altered fluid volumes and increased renal blood flow in these patients are the main factors responsible for pharmacokinetic changes that require higher doses, reduction on time dose intervals or both of them.

## Objective

Anti-infective plasma measurements in one burn patient with sepsis to determine whether drug efficacy was achieved, thereby improving the likelihood of infection control.

## Methods

A male burn child, 8 years old, 40 kg with severe thermal plus inhalation injuries (petrol), 45% total burn surface area, was investigated. He has received six anti-infective agents during the 88-day period in the ICU. Drug plasma monitoring, pharmacokinetics and the PK-PD correlation were done by blood sample collection, and drug plasma measurements were performed by high-performance liquid chromatography.

## Results

Since in burns pharmacokinetics is unpredictable for all agents investigated, drug efficacy was based on PK-PD correlation (Table 1). Dose adjustment was performed for vancomycin (from 0.5 g 6-hourly to 1 g 8-hourly), meropenem (from 0.75 to 1 g 8-hourly) and linezolid (from 0.3 to 0.6 g 12-hourly).

**Table 1:** Pharmacokinetics and PK-PD correlation for six anti-infective agents for a burn child [AQ2]

		Drug efficacy (%)^a^/*MIC *(mg/l)^b^			*t*_1/2 _(hours)		CL (ml/minute/kg)		Vd (l/kg)	
**Drug**	**Follow-up periods**				**Obtained**	**Reference^c^**	**Obtained**	**Reference^c^**	**Obtained**	**Reference^c^**

Fluconazole	1	100%	100%	100%	22.10	27 to 37	0.27	0.20 to 0.34	0.53	0.50 to 0.70
		*8 mg/ml*	*16 mg/ml*	*32 mg/ml*						
Imipenem	3	100%	100%	100%	1.90	0.8 to 1.0	4.62	2.6 to 3.1	0.76	0.18 to 0.28
		*0.5 mg/ml*	*1 mg/ml*	*4 mg/ml*						
Linezolid	2	100%	50%	0%	3.45	4.5 to 5.4	3.22	1.14 to 2.08	0.95	0.57 to 0.86
		*1 mg/ml*	*2 mg/ml*	*4 mg/ml*						
Meropenem	8	100%	100%	62%	2.00	1.0	3.81	2.7 to 4.3	0.60	0.17 to 0.28
		*0.5 mg/ml*	*2 mg/ml*	*8 mg/ml*						
Sulphamethoxazole	2	50%	50%	ND	19.65	7.5 to 12.7	0.74	0.24 to 0.38	1.34	0.22 to 0.30
		*32 mg/ml*	*64 mg/ml*	*ND*						
Vancomycin	5	80%	60%	60%	2.10	5.0 to 11.0	1.46	1.3 to 1.5	0.30	0.33 to 0.45
		*1 mg/ml*	*2 mg/ml*	*4 mg/ml*						

^a^Parameters PK-PD for *in vivo*-*in vitro *correlation: AUC^ss^_0 to 24_/MIC or %T >MIC. ^b^Eucast, 2011. ^c^Goodman and Gilman, 2006; Micromedex, 2010.

## Conclusion

PK-PD correlation was applied to investigate changes on dose regimen to reach the efficacy for all anti-infective agents. Dose adjustments were required only for vancomycin, linezolid, and meropenem to guarantee drug efficacy.

